# Supplementary material for: Chromothripsis during telomere crisis is independent of NHEJ, and consistent with a replicative origin
Source: Genome Res. 2019 May;29(5):737–49. doi: 10.1101/gr.240705.118 (PMC6499312; doi:10.1101/gr.240705.118)
Supplement: Supplemental Material [file supp_gr.240705.118_Supplemental_file_1.zip › contigs/annotated_contigs/DB53/contig.2.DB53_length_288_mean_cov_12.28125.docx]

**DB53_length_288_mean_cov_12.28125**

```
ACAGACACAA|AAAGAGACACAGAGAGAAAGACAGAGATGAACAGAGAGAGACAGAGAGAAACAGACAGAGAGAAGGTCCTAGCCCAGT
           >chr2:133029279-133029405 + E=4e-47 p=2e-02
AGCGATACAGTGCCTTTTCTTTCATTTTCTCTTTCTTTTCTTTT|TGTCTTTCTTT|TATGTATGTATGTATGTATGTATGTATGTATG
                                                        >chrUn_gl000220:130905-131050 -
TATGTATTTATCTGGAGACCGGGTCTCACTCTGTCACCCAGGCTGTAGTGCAGTGGCACGATCTTGGGTCACTGCAACCTCCGCCTGCC
 E=7e-52
AGGTTCAAGCAATTCTTCCACCCC
```
